# Predictive Factors for Successful Type 1 Big Bubble during Deep Anterior Lamellar Keratoplasty

**DOI:** 10.1155/2018/4685406

**Published:** 2018-11-13

**Authors:** Vincent M. Borderie, Sara Touhami, Cristina Georgeon, Otman Sandali

**Affiliations:** Centre Hospitalier National d'Ophtalmologie des 15-20, Sorbonne Université, Paris, France

## Abstract

**Objective:**

Big bubble (BB)-deep anterior lamellar keratoplasty (DALK) has become the reference transplantation technique for corneal stromal disorders. Type 1 BB is the desired aspect but it is not constant. We aimed to determine the predictive factors of type 1 BB success.

**Methods:**

Observational cohort study including 77 consecutive eyes of 77 patients undergoing DALK by one surgeon at a single reference center without any selection. Clinical and spectral domain optical coherence tomography data were collected pre- and postoperatively.

**Results:**

Stromal scars were found in 91.8% of cases and were located in the anterior (90.9%), mid (67.5%), and posterior (36.4%) stroma. Type 1 BB (49.3% of cases) was significantly associated with the absence of scars in the posterior stroma, stage 1–3 keratoconus, and deep trephination. Among eyes with posterior scars, type 1 BB was associated with higher minimal corneal thickness, maximum-minimum corneal thickness < 220 *μ*m, and diagnosis other than keratoconus. Eyes with type 1 BB featured significantly thinner residual stromal bed (22 ± 8 *µ*m versus 61 ± 28 *µ*m), thinner corneas at 12, 24, and 36 months, and better visual acuity at 12 months compared with eyes with no type 1 BB. Conversely, no significant differences between both groups were observed for graft survival, visual acuity at 24 and 36 months, and endothelial cell density at 12 and 36 months.

**Conclusion:**

OCT assessment before DALK is useful for choosing trephination depth that should be as deep as possible and for looking for posterior scars. The BB technique may not be the most appropriate method in keratoconus with posterior scars. Follow-up data do not support the need for conversion to penetrating keratoplasty when type 1 BB cannot be obtained nor does it support the need for performing a penetrating keratoplasty as a first-choice procedure in eyes with posterior stromal scars.

## 1. Introduction

Deep anterior lamellar keratoplasty (DALK) is nowadays the first-choice operative technique for corneal diseases not involving the endothelium because it offers advantages over penetrating keratoplasty including preservation of the recipient's endothelium [1–9]. Amongst the factors that influence visual outcome after DALK, quality of donor/host interface and amount and regularity of residual stroma adherent to Descemet membrane are probably the most important [10]. Minimizing the residual stroma (<65 microns) was shown to provide good visual outcomes that are similar to those obtained with penetrating keratoplasty [11]. The development of surgical methods that allow removing the diseased stroma with greater efficacy has led to a renewed interest in lamellar keratoplasty. However, techniques that have attempted to bare Descemet membrane resulted in significant perforation rates and increased complications [12]. One of the safest and most popular methods to obtain maximum depth DALK is the big bubble technique that allows cleavage at the level of a predescemetic plane, leaving only a thin layer of residual stroma (i.e., Dua's layer) above the Descemet membrane [10, 13]. This technique consists in a forceful injection of air into the deep stroma after partial thickness trephination which creates an intracorneal bubble that can take 2 forms. In type 1 big bubble, the desired result, air forms a well-circumscribed bubble located between Dua's layer and the remaining stroma, up to 8.5 mm in diameter, starting at the center and expanding progressively to the periphery [13]. Partial thickness anterior keratectomy can then leave a thin layer of stroma anterior to the bubble. An iris spatula can be introduced into the space left by the collapsed bubble, and the remaining stroma can be excised. Type 2 big bubble is a large thin-walled bubble. It starts in the peripheral cornea and enlarges very quickly centrally. It is located between Dua's layer and Descemet membrane. In such cases, further surgical steps are at high risk of perforation due to very thin posterior recipient bed.

DALK has obvious advantages over penetrating keratoplasty at the cost of a steep learning curve, which the big bubble technique has facilitated. However, type 1 big bubble is not constant even for experienced surgeons and manual dissection with a risk of perforation is often necessary when type 1 bubble fails. The objectives of our study were to determine the predictive factors for type 1 big bubble success based on simple clinical and OCT parameters and to compare the outcome of DALK in eyes with successful type 1 big bubble with that of DALK in eyes with failed type 1 big bubble.

## 2. Materials and Methods

This hospital-based observational cohort study was conducted at the French National Eye Hospital (Centre Hospitalier National d'Ophtalmologie des 15–20, Paris, France). Institutional review board approvals for chart reviews were obtained commensurate with the respective institutional requirements prior to the beginning of the study. Described research was approved by the Ethics Committee of the French Society of Ophthalmology and adhered to the tenets of the Declaration of Helsinki. Informed consent was obtained for all patients. All consecutive patients with stromal disease requiring keratoplasty between January 2013 and January 2015 were included. They all underwent DALK as a first-choice operative technique whatever the stromal condition was.

Inclusion criteria were the following: DALK performed by one surgeon (V. M. B.) for optical reasons in eyes with corneal disease not involving the endothelium and pre- and postoperative assessment with spectral-domain optical coherence tomography. During the study period, all eyes with stromal disorders and normal corneal endothelial function (i.e., absence of corneal edema on slit lamp examination) were considered for DALK whatever their endothelial cell density is. Data were recorded prospectively and analyzed retrospectively. For each patient, demographic, clinical, and high-resolution optical coherence tomography (RTVue^R^; Optovue, Inc Fremont, CA) data were collected preoperatively and included corneal disease, best spectacle-corrected visual acuity (BCVA) (LogMAR visual acuity), central and minimal corneal thicknesses, central and minimal epithelial thicknesses, difference between maximal and minimal corneal thickness, and location of stromal scars on OCT scans. When the disease was keratoconus, the disease stage according to OCT classification (Table 1) was recorded [14]. Location of stromal opacity on OCT scans was made according to the maximum depth of opacity, with stroma divided into three equally thick zones (i.e., anterior stroma, midstroma, and posterior stroma). Peroperatively, diameter and depth of trephination were recorded.

Table 1 is reproduced from Sandali et al. (under the Creative Commons Attribution License/public domain) [14].

All surgeries were performed under general anesthesia, using the “big bubble” technique as previously described [15]. Briefly, the first steps included partial thickness trephination of variable depth depending on the peripheral corneal thickness using the Hanna trephine (Moria, Antony, France). The trephination diameter was chosen preoperatively based on the white-to-white corneal diameter. The 7–10 mm corneal zone of the SD-OCT pachymetry map was used to choose the trephination depth. The trephination depth was made as deep as possible with a security margin of 100 *µ*m above the thinnest point of the peripheral 7–10 mm corneal zone. Then, partial anterior keratectomy was performed with a crescent blade. A 30 gauge hypodermic needle faced bevel down was then used for air injection at the base of the trephination gutter to obtain a “big bubble.” Type 1 big bubble definition was single well-circumscribed central dome-shaped bubble starting from the center and extending progressively towards the periphery, so that it could be stopped at the level of the trephination gutter. When no type 1 big bubble was obtained, despite repeated air injections, a manual dissection technique was performed as previously reported [15].

Follow-up data were recorded including graft transparency, best spectacle-corrected visual acuity, central corneal thickness measured with high-resolution optical coherence tomography, and endothelial cell density measured with wide-field specular microscopy (Topcon, Clichy, France) as part of routine care.

The main outcome measure was the occurrence of a type 1 big bubble. All variables were correlated with the occurrence of a type 1 big bubble to identify its predictive factors. In case of type 2 big bubble formation, manual dissection was performed. These eyes were included in the “no type 1 big bubble” group.

Statistical analyses' results were presented as mean ± standard deviation (SD) for continuous variables and as proportions (%) for categorical variables. A Kaplan–Meier plot was used to assess graft survival. Stratified Cochran chi-square, Student, log-rank, and Fisher exact tests were used for intergroup comparisons when appropriate. *p* values of 0.05 or less were considered significant. Analyses were performed using a software program (Statistica version 6.1; StatSoft France, Maisons-Alfort, France).

## 3. Results

Seventy-seven eyes of 77 patients met the inclusion criteria. Characteristics of patients, preoperative OCT assessment, and surgical data are shown in Table 2. Keratoconus patients were younger than the patients with other corneal disorders (mean age: 34.1 years in the keratoconus patients versus 51.3 years in the nonkeratoconus patients; *p*=0.0008). The female-to-male sex ratio was 1. Corneal scars were found in 91.8% of cases on OCT and were located at the level of the anterior, mid, and posterior stroma in, respectively, 90.9%, 67.5%, and 36.4% of cases (Table 2).

Type 1 big bubble was obtained in 38 of 77 eyes (49.3%). No perforations occurred in eyes with type 1 big bubble. Among the 39 eyes (50.7%) with no type 1 big bubble, dissection was completed manually and perforation (including micro- and macroperforations) occurred in 7 eyes during deep stromal dissection. When a perforation occurred, the perforated zone was localized, and no further dissection was performed in this zone. The anterior chamber was filled with air, and cautious dissection was continued outside this zone. Overall, 100% of the patients with perforations had posterior scars. Besides, 100% of the perforations occurred in keratoconus patients, and none required conversion to penetrating keratoplasty.

Occurrence of type 1 bubble was associated with absence of posterior scars in preoperative OCT, stages 1 to 3 keratoconus (versus stage 4 or 5), and deep trephination (Table 2). Corneas with stage 4 or 5 keratoconus feature presence of posterior scars in all cases. Type 1 big bubble was achieved in 65% of eyes with no posterior scars and 21% of eyes with posterior scars. Several parameters showed no significant association with type 1 big bubble formation including patient age, preoperative visual acuity, corneal endothelial cell density, OCT central epithelial thickness, presence of scars in the anterior or midstroma on OCT scans, and trephination diameter (Table 2).

Among 28 eyes with posterior scars on OCT, type 1 big bubble was associated with higher minimal corneal thickness, higher minimal epithelial thickness, difference between maximum and minimum corneal thickness below 220 *µ*m, and diagnosis other than keratoconus (Table 3). Type 1 big bubble was obtained in 8% of keratoconus versus 100% of nonkeratoconus eyes with posterior scars.

Table 4 shows the postoperative outcomes in both groups. The thickness of the residual stromal bed was 22 ± 8 *µ*m (mean ± standard deviation) in eyes with type 1 big bubble and 61 ± 28 *µ*m in eyes with no type 1 big bubble (*p* < 0.0001). Eyes with type 1 big bubble featured significantly thinner corneas at 12, 24, and 36 months and better visual acuity at 12 months compared with eyes with no type 1 big bubble. Conversely, no significant differences between both groups were observed for graft survival, visual acuity at 24 and 36 months, and endothelial cell density at 12 and 36 months.

## 4. Discussion

Despite its obvious advantages, the extensive learning duration and technical difficulty of DALK still lead many surgeons to prefer penetrating keratoplasty [11–13]. If it is nowadays commonly admitted that big bubble DALK is an efficient method to obtain deep and efficient keratectomies, other lamellar dissection techniques are possible, but they present one major flaw that is the absence of visualization of dissection depth which can result in perforation [12, 16–24]. This can be limited by the use of peroperative OCT; however, the latter is not always available [25, 26]. In this context, the big bubble technique seems to allow maximum depth keratoplasty with low perforation rates especially when type 1 big bubble is obtained. Dua's layer baring DALK was shown to withstand higher intraoperative pressures than Descemet's membrane baring DALK [27]. Besides, this technique seems to be more efficient in baring Dua's layer/Descemet membrane than other methods [24–26, 28]. Nevertheless, the likelihood of type 1 big bubble is not 100%, even in the hands of experienced surgeons, which advocates for the need for objective factors to predict type 1 big bubble occurrence [29, 30]. Our study was designed to show the value of easily available preoperative parameters to predict the success of a type 1 big bubble. We found that the occurrence of type 1 big bubble was significantly associated with absence of posterior scars on OCT, stages 1 to 3 keratoconus, and deep trephination.

While we found no apparent correlation with age, this negative statement provides interesting knowledge about corneal histology and its natural history. In fact, we showed that the average thickness of the recipient's residual stromal bed was 22 ± 8 *µ*m in eyes with successful type 1 big bubble, which is slightly higher than combined Descemet membrane and endothelium thicknesses. This confirms that type 1 dissection must occur at the level of the predescemetic Dua's layer [13]. The fact that we did not find any correlation between age and type 1 big bubble occurrence suggests that this predescemetic layer might not be subjected to biochemical or biomechanical changes with aging contrarily to the Descemet membrane. Conversely for Descemet's membrane endothelial keratoplasty graft preparation, physiologic cleavage plane between the interfacial matrix, the anterior most adhesive zone of Descemet membrane, and stroma is used, and it has been shown to feature interindividual and age-related variations in structure and composition [31].

Presence of scars at the anterior and midstroma did not influence the occurrence of type 1 big bubble since air was directly injected into the deep stroma [10]. Conversely, presence of posterior scars (corresponding to stage 3b, 4, or 5 keratoconus on OCT (Table 1)) could disrupt bubble progression leading to failed type 1 aspect. The depth of trephination probably prevents the air from reaching the peripheral cornea and migrating though the trabecular meshwork to the anterior chamber which could facilitate access to the predescemetic layer explaining the high rates of type 1 bubbles observed with deep trephinations. However, the relationship between trephination depth and type 1 BB formation could simply result from the injection of air nearest of the deep layer.

The most interesting point of this study was that the presence of posterior scars was a strong predictive factor for type 1 big bubble failure which is consistent with results reported by other investigators [32]. When looking specifically at the correlations in the subpopulation with posterior scars on OCT, we found that type 1 big bubble was significantly associated with higher minimal corneal thickness, higher minimal epithelial thickness, difference between maximum and minimum corneal thickness lower than 220 *µ*m, and diagnosis other than keratoconus. In fact, thin corneas make it more difficult to control trephination depth and needle position when deep intrastromal air injection is intended. This is particularly relevant in case of posterior scars. Peroperative OCT should make these steps easier to achieve in such cases. In addition, corneas featuring very thin stroma with posterior scars might present significant damage or fibrosis of the predescemetic layer that could prevent cleavage between the latter layer and the former underlying stroma. Interestingly, in case of posterior scars, corneal disease seemed to be a predictive factor of type 1 big bubble success. We found that keratoconus was a risk factor for type 1 big bubble failure in the presence of posterior scars. This suggests that keratoconus probably induces different posterior scarring features compared with other diseases with different biomechanical behaviors as suggested by different characteristics of stromal striae in keratoconus eyes compared with other corneal disorders [33]. Keratoconus probably makes the corneal structure airtight and therefore type 1 big bubble aspect more likely to fail. Besides, it is possible to speculate that thinning process in keratoconus is probably more heterogeneous than in other diseases, which makes trephination and dissection more hazardous. For patients with a history of hydrops (i.e., stage 5 keratoconus), various surgical approaches can be considered. Usually, a PK is performed in order to get the best visual recovery. A DALK is still possible using various techniques [34, 35]. If an air injection is performed, it has to be done cautiously mainly to facilitate deep stromal dissection.

In routine practice, corneas with posterior stromal scarring are usually considered for PK and not for DALK. Conversely, we always schedule DALK in patients with stromal disorders and maintained endothelial function whatever the posterior stromal condition based on the mid- and long-term advantages of DALK over PK is. The drawback is that the rate of type 1 BB formation is lower than expected.

Our results seem to converge towards one important statement: the population of keratoconus patients with posterior scars and thin corneas seems to be a subgroup of interest that should need special attention and extensive preparation before DALK. This is important because it could induce interesting changes in the management of the patients. On one hand, presence of anterior hyperreflective opacities at Bowman's layer level, epithelial thickening, and stromal thinning at the conus without posterior scarring (corresponding to stage 3a on OCT) are strong risk factors for hydrops [14, 36]. None of stage 4 patients develop hydrops. It should be wise to always intend big bubble DALK in the absence of deep posterior scars, and in keratoconus cases, to propose this surgery as early as stage 3a (in case of impaired BCVA) in order to reduce both the risks of hydrops and that of making big bubble DALK hazardous [11]. On the other hand, in keratoconus with deep posterior scars, it probably would be wiser to perform manual dissection assisted by peroperative OCT, rather than taking the risk of perforation with the big bubble technique. Other dissection techniques such as Melles' method, manual dissection, hydrodissection, and viscodissection could be intended [37–39].

A large trephination size has been reported to be associated with an increase in the probability of successful big bubble formation in keratoconus eyes [40].

Postoperative follow-up of our patients showed that eyes with type 1 big bubble had faster visual recovery and thinner corneas than eyes with no type 1 big bubble. Three years after DALK, both groups had similar graft survival, endothelial cell density, and visual recovery. Failed type 1 big bubble has probably minimal consequences for the long-term results of DALK when the procedure is achieved with the manual dissection technique with no conversion to penetrating keratoplasty.

**Figure 1:**
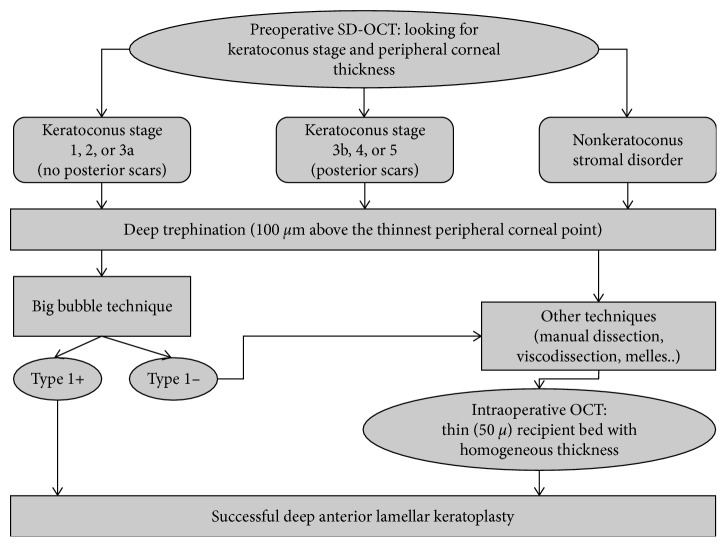
An algorithm for performing DALK according to preoperative optical coherence tomography (OCT) assessment of recipient cornea.

Figure 1 shows an algorithm for performing DALK, taking advantage of preoperative OCT assessment of recipient cornea and intraoperative OCT-assisted surgery derived from the results of the present study.

There are a few limitations to the present study that need to be addressed. First, the study was not conducted in a prospective interventional way, and our results must be moderated by the fact that a type 2 error cannot be dismissed. Second, our type 1 bubble success rate was slightly below the reported figures [41]. However, our cohort was probably more severe than what was reported in the literature because all patients with stromal disorder and normal endothelial function were considered for DALK. In fact, most eyes had stromal opacities on preoperative OCT, whereas big bubble DALK is usually used in eyes with no scars. Another explanation could be related with the big bubble DALK technique we used. We performed anterior keratectomy prior to intrastromal air injection, which could reduce chances of getting type 1 big bubble. Comparison between anterior stromal removal before or after air injection needs to be further studied. At last, use of smooth cannula to inject air has been reported to result in more type 1 BB formation compared with use of needle [42]. Currently, we have modified our surgical technique by injecting air under peroperative OCT control before the anterior keratectomy.

## 5. Conclusions

OCT assessment of eyes before DALK appears to be useful for choosing trephination depth that should be as deep as possible and for looking for scars at the level of the posterior stroma. Surgical technique should be adapted in keratoconus with posterior scars, and the big bubble technique may not be the most appropriate method in these eyes, although still possible. Whether the procedure has to be started directly with another lamellar technique or with the big bubble technique with further conversion to another lamellar technique requires further investigations. Data obtained from 3-year follow-up of patients do not support the need for conversion to penetrating keratoplasty when type 1 big bubble cannot be obtained nor does it support the need for performing a penetrating keratoplasty as a first-choice procedure in eyes with posterior stromal scars.

## Figures and Tables

**Table 1:** Optical coherence tomography classification of keratoconus.

Stage	Characteristics
1	Thinning of epithelial and stromal layers at the conus. Corneal layers have a normal aspect.
2	Hyperreflective anomalies occurring at Bowman's layer level and epithelial thickening at the conus:
	2a: Clear stroma.
	2b: Stromal opacities.
3	Posterior displacement of the hyperreflective structures occurring at Bowman's layer level with increased epithelial thickening and stromal thinning:
	3a: Clear stroma.
	3b: Stromal opacities.
4	Pan-stromal scar.
5	Hydrops stage:
	5a: Acute onset, characterized by the rupture of Descemet's membrane with delamination of collagen lamellae, large fluid-filled intrastromal cysts, and the formation of epithelial edema.
	5b: Healing stage, pan-stromal scarring with a remaining aspect of Descemet's membrane rupture.

**Table 2:** Characteristics of patients and surgical procedures.

Variable	Overall (*n*=77)		Type 1 big bubble (*n*=38)	No type 1 big bubble (*n*=39)	*p* value
	Mean/number	Range/percentage	Mean/number	Mean/number	
Patient age (years)	37.9	[13; 77]	39.3	36.6	0.43
Preoperative diagnosis:					**0.0002**
** **Keratoconus and other ectatic disorders	62	80.5%	27	35	
** **Scar after infectious keratitis	6	7.8%	4	2	
** **Stromal dystrophy	6	7.8%	5	1	
** **Scar after trauma	2	2.6%	2	0	
** **Stromal opacification after penetrating Keratoplasty	1	1.3%	0	1	
Preoperative best-corrected LogMAR visual acuity	1.23	[0.5; 2.3]	1.21	1.24	0.81
OCT central epithelial thickness (*µ*m)	49	[38; 66]	49	49	0.89
OCT minimal epithelial thickness (*µ*m)	29	[8; 55]	30	28	0.57
OCT central corneal thickness (*µ*m)	418	[144; 682]	425	412	0.59
Keratoconus OCT classification (Table 1):					**0.0001**
** **Stage 1	3	4.8%	1	2	
** **Stage 2	13	21.0%	7	6	
** **Stage 3	22	35.5%	17	5	
** **Stage 4	23	37.1%	2	21	
** **Stage 5	1	1.6%	0	1	
Scar in posterior stroma:					**0.0002**
** **Yes	28	36.4%	6	22	
** **No	49	63.6%	32	17	
Scar in midstroma:					0.12
** **Yes	52	67.5%	23	29	
** **No	25	32.5%	15	10	
Scar in anterior stroma:					0.72
** **Yes	70	90.9%	35	35	
** **No	7	9.1%	3	4	
Maximal-minimal corneal thickness > 220 *µ*m (*n*=75):					0.13
** **Yes	43	57.3%	18	25	
** **No	32	42.7%	19	13	
Preoperative recipient corneal endothelial cell density (cells/mm^2^)	2654	[1000; 3450]	2631	2689	0.75
Trephination diameter (mm)	8.17	[7.5; 8.5]	8.18	8.17	0.74
Trephination depth (*µ*m)	421	[250; 550]	435	408	**0.03**

OCT: optical coherence tomography. Data were available for the 77 patients unless indicated.

**Table 3:** Correlation analysis for eyes with posterior scars on preoperative optical coherence tomography (OCT) assessment (*n*=28).

Variable	*p* value
OCT minimal epithelial thickness (*µ*m)	**0.003**
OCT central epithelial thickness (*µ*m)	0.89
OCT minimal corneal thickness (*µ*m)	**0.03**
OCT central corneal thickness (*µ*m)	0.16
Maximal-minimal corneal thickness < 220 (um)	**0.006**
Trephination diameter (mm)	0.44
Trephination depth (*µ*m)	0.06
Diagnosis other than keratoconus	**0.007**

**Table 4:** Postoperative outcome of surgical procedures.

Variable	Overall (*n*=77)		Type 1 big bubble (*n*=38)	No type 1 big bubble (*n*=39)	*p* value
	Mean/estimate^*∗*^	Range/95% confidence interval^*∗*^	Mean/estimate^*∗*^	Mean/estimate^*∗*^	
Follow-up time (months)^*∗∗*^	46	[1; 66]	47	44	0.77
36-month graft survival (%)	97	[93; 100]	95	100	0.16
LogMAR best-corrected visual acuity:					
** **12 months after DALK (*n*=72)	0.36	[0.00; 1.30]	0.30	0.41	**0.036**
** **24 months after DALK (*n*=65)	0.30	[0.00; 1.10]	0.26	0.35	0.11
** **36 months after DALK (*n*=65)	0.28	[0.00; 1.30]	0.23	0.34	0.10
Central corneal thickness (*µ*m):					
** **12 months after DALK (*n*=59)	538	[425; 680]	509	567	**<0.0001**
** **24 months after DALK (*n*=61)	557	[460; 790]	536	576	**0,004**
** **36 months after DALK (*n*=61)	558	[475; 745]	546	573	**0,026**
Corneal endothelial cell density (cells/mm^2^):					
** **12 months after DALK (*n*=60)	2310	[550; 3100]	2245	2380	0.38
** **36 months after DALK (*n*=43)	2222	[650; 3100]	2245	2195	0.75

OCT: optical coherence tomography. ^*∗*^For survival data. ^*∗∗*^From keratoplasty to last visit for successful grafts and from keratoplasty to graft failure for the failed graft. Data were available for the 77 patients unless indicated.

## Data Availability

The data used to support the findings of this study are available from the corresponding author upon request.
